# Deciphering the ovarian cancer ascites fluid peptidome

**DOI:** 10.1186/1559-0275-11-13

**Published:** 2014-04-02

**Authors:** Anand Bery, Felix Leung, Christopher R Smith, Eleftherios P Diamandis, Vathany Kulasingam

**Affiliations:** 1 Department of Laboratory Medicine and Pathobiology, University of Toronto, Toronto, ON, Canada; 2 Department of Clinical Biochemistry, University Health Network, Toronto, ON, Canada; 3 Department of Pathology and Laboratory Medicine, Lunenfeld-Tanenbaum Research Institute, Mount Sinai Hospital, Toronto, ON, Canada

**Keywords:** Biomarker, Early diagnosis, Mass spectrometry, Ovarian cancer, Ascites fluid, Peptidome

## Abstract

**Background:**

Conventional proteomic approaches have thus far been unable to identify novel serum biomarkers for ovarian cancer that are more sensitive and specific than the current clinically used marker, CA-125. Because endogenous peptides are smaller and may enter the circulation more easily than proteins, a focus on the low-molecular-weight region may reveal novel biomarkers with enhanced sensitivity and specificity. In this study, we deciphered the peptidome of ascites fluid from 3 ovarian cancer patients and 3 benign individuals (ascites fluid from patients with liver cirrhosis).

**Results:**

Following ultrafiltration of the ascites fluids to remove larger proteins, each filtrate was subjected to solid phase extraction and fractionated using strong cation exchange chromatography. The resultant fractions were analyzed using an Orbitrap mass spectrometer. We identified over 2000 unique endogenous peptides derived from 259 proteins. We then catalogued over 777 peptides that were found only in ovarian cancer ascites. Our list of peptides found in ovarian cancer specimens includes fragments derived from the proteins vitronectin, transketolase and haptoglobin.

**Conclusions:**

Peptidomics may uncover previously undiscovered disease-specific endogenous peptides that warrant further investigation as biomarkers for ovarian cancer.

## Background

The advent of high-throughput, mass spectrometry-based proteomics for biomarker discovery was met with optimism over a decade ago. The ability to accurately capture and catalogue numerous proteins in a given sample was thought to hold great potential, and many felt that next-generation biomarkers for various cancers would be in the clinic within a few years. Unfortunately, conventional “bottom-up” proteomics has thus far been unable to uncover novel serum-based markers that are more sensitive and specific than existing markers such as prostate-specific antigen for prostate cancer and carbohydrate antigen 125 (CA-125) for ovarian cancer (OvCa) [1]. Conventional proteomic approaches have struggled to overcome a number of inherent limitations to biomarker discovery, namely the low relative abundance of potential markers (which often exist in concentrations up to ten million-fold lower than circulating high-abundance proteins [2]), and the enormous heterogeneity of secreted proteins across samples, sub-populations, and disease sub-types [3,4].

Ultimately, the difficulties that conventional proteomics has faced in addressing these confounds necessitated the exploration of alternate approaches to biomarker discovery. The focus of several research groups has recently turned to the low-molecular-weight (LMW) region, or peptidome, of biological fluids. Previously billed as a “treasure trove of diagnostic potential” [5], this region represents a rich source to mine for new biomarkers. The rationale is that proteolytic degradation products will make their way from interstitial fluid, to lymph, to the bloodstream (where they would ultimately be assayed) more easily than their larger protein counterparts [6,7]. Since proteolytic activity likely increases with the progression of malignancy, we expect to find a wealth of degradation products in the cancerous state [6]. Progression of malignancy is also associated with the degradation of adhesion and cell-to-cell junction proteins, and proteolytic products of these proteins are likely to be differentially expressed in cancer, both intracellularly and in the circulation, thereby marking the disease state.

Past studies have already demonstrated the utility of peptidomic approaches. Villanueva et al. found that a specific signature of serum peptides was able to distinguish patients with 3 different types of solid tumours from those without cancer [8]. The group suggested that exoprotease activity contributes to the generation of cancer-specific (and even cancer type-specific) peptides in serum. More recently, Fiedler et al. employed peptidomic profiling in a search for pancreatic cancer markers, identifying two mass spectrometric peaks that were able to distinguish patients from healthy controls with a sensitivity of 86.3% and a specificity of 97.6% [9].

In this study, we applied a novel peptidomics approach to ascites fluid originating from OvCa. Our work joins a small but growing body of existing studies on the use of peptides for OvCa diagnostics. Lopez et al. found several peptide biomarker panels that could differentiate stage 1 ovarian cancer patients from age-matched controls [10]. Similarly, Fredolini et al. reported 59 peptide markers differentially expressed in OvCa patients compared to patients with benign gynecological conditions [2]. Unlike the above-mentioned studies, which examined the peptidome of serum, our study mined ascites fluid, an excellent choice for biomarker discovery work. Since ascites fluid accumulation occurs close to the target tissue (proximity to disease is high), the fluid contains many cells of tumour origin, as well as soluble factors released by both tumour cells and the local microenvironment. Microenvironmental factors are important since putative biomarkers can originate from interactions between the tumour and the surrounding environment as well as from the cancerous tissue itself.

Despite significant research gains, there continues to be an urgent need for novel biomarkers of ovarian cancer. Early detection can close the gap between diagnosis at early stages (which carries a five-year survival rate of up to 95%) and diagnosis at later stages (with a five-year survival rate of 10-30%) [11]. The existing clinically accepted serum marker for ovarian cancer is CA-125, which, despite its widespread use for monitoring therapeutic response, is not sensitive or specific enough for early diagnosis. Other studied markers, including HE4, osteopontin, CA 15–3, CA 19–9, and various human tissue kallikreins, show utility in detecting ovarian cancer, but none are effective for screening or early diagnosis [12,13]. The low-molecular-weight proteome might uncover previously undiscovered alternatives worthy of investigation. Our study identified over one thousand peptides in ovarian cancer ascites (many of which were expressed uniquely in cancer) that warrant further verification and validation in future studies.

## Results and discussion

### Peptidome identification

**Figure 1:**
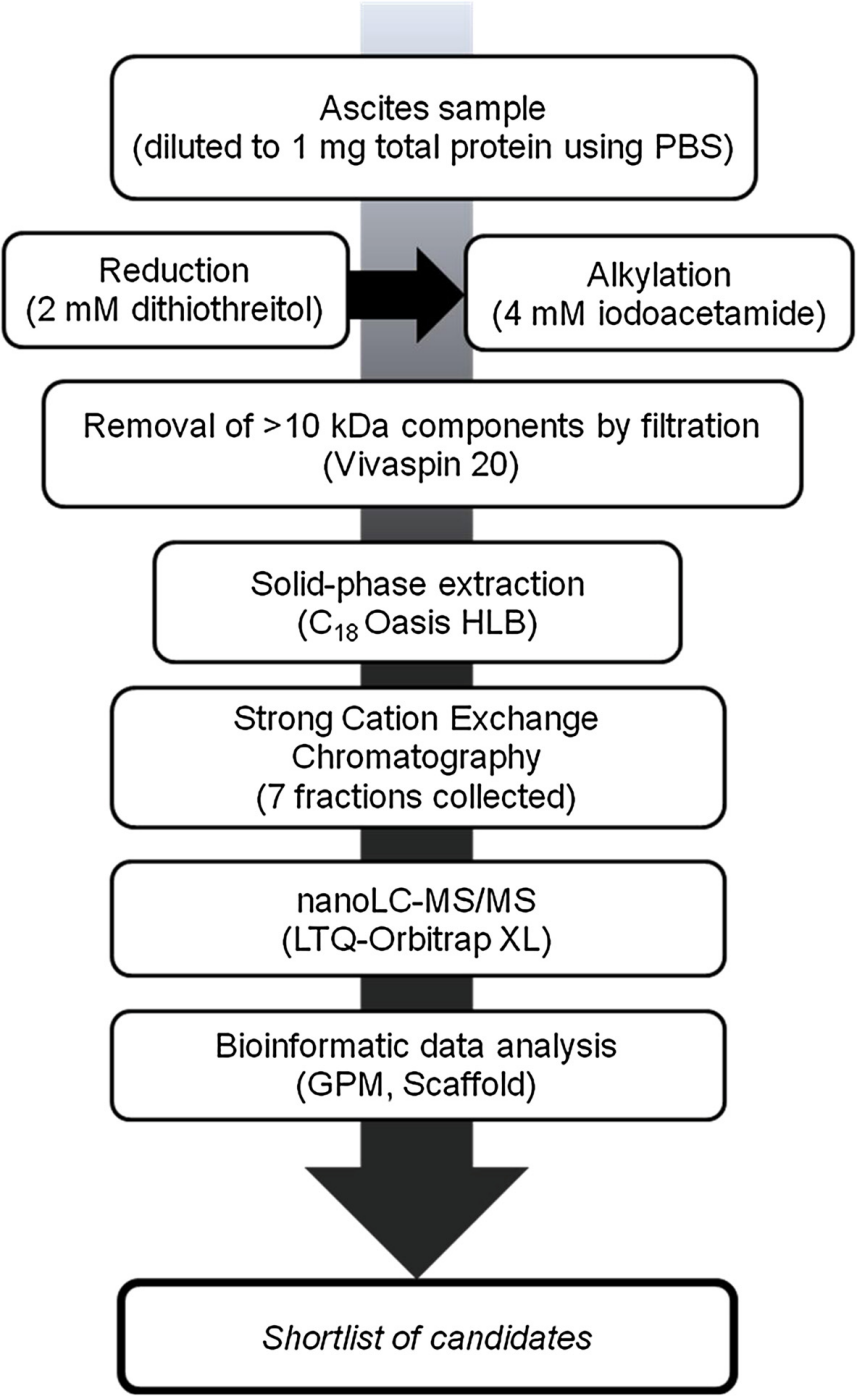
**Outline of experimental workflow (peptidomic analysis).** The workflow consisted of the following: sample processing, followed by strong cation exchange and reverse-phase chromatography coupled online to an LTQ-Orbitrap mass spectrometer and subsequent data analysis.

**Figure 2:**
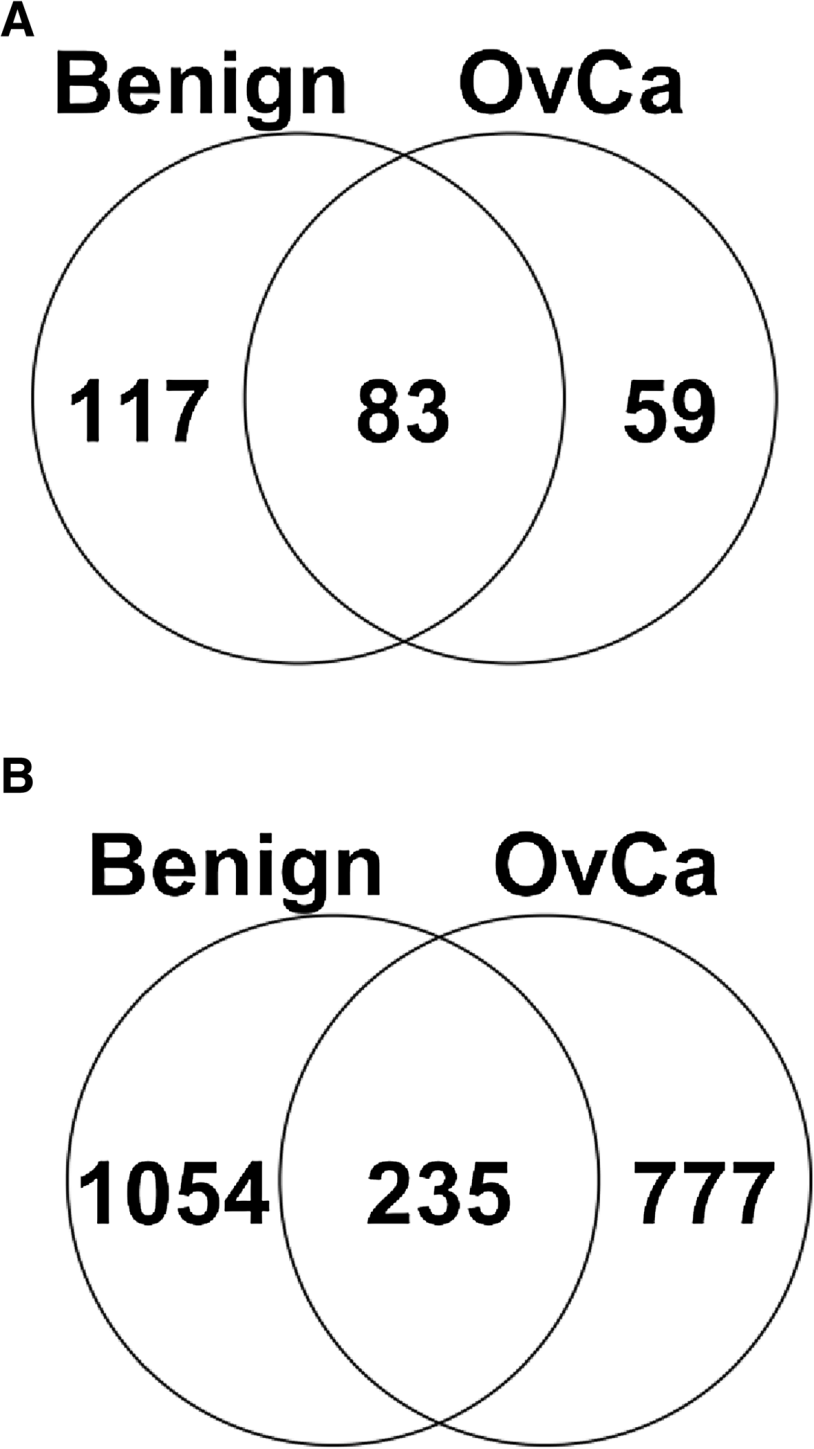
**Overlap of the proteins (A) and peptides (B) identified in benign (n = 3) and ovarian cancer (OvCa) (n = 3) ascites samples.** Fifty-nine unique proteins were found in the OvCa ascites fluid and 777 peptides were unique to OvCa ascites compared to benign controls (ascites fluid from liver cirrhosis patients).

Using the experimental workflow depicted in Figure 1, we elucidated the endogenous peptidome of 6 ascites fluid samples, 3 from OvCa patients and 3 from non-cancer controls. This is, to our knowledge, the first study to mine the low-molecular-weight region of OvCa ascites. We identified 2066 unique peptides (Additional file 1), 777 of which were found in OvCa but not in the control samples. The 777 OvCa-specific peptides corresponded to only 59 proteins, indicating that many of the peptides identified are degradation products of the same parent proteins. The list of the 59 parent proteins and the corresponding 777 unique peptides is provided in Additional file 2A and B. A full spectrum report, which includes information about modifications associated with each peptide, is provided in Additional file 3. Figure 2 (A) and (B) display the overlap of identified proteins and peptides between the benign and OvCa samples, respectively. Interestingly, our study identified a larger number of peptides from benign samples than from OvCa samples, suggesting that protease activity alone may not be sufficient to discriminate cancerous from benign ascites fluid. Our benign ascites fluid originated from patients with cirrhosis of the liver, a condition associated with a rise in both inflammatory mediators and matrix metalloproteases [14-17].

### Reproducibility across all six samples

**Figure 3:**
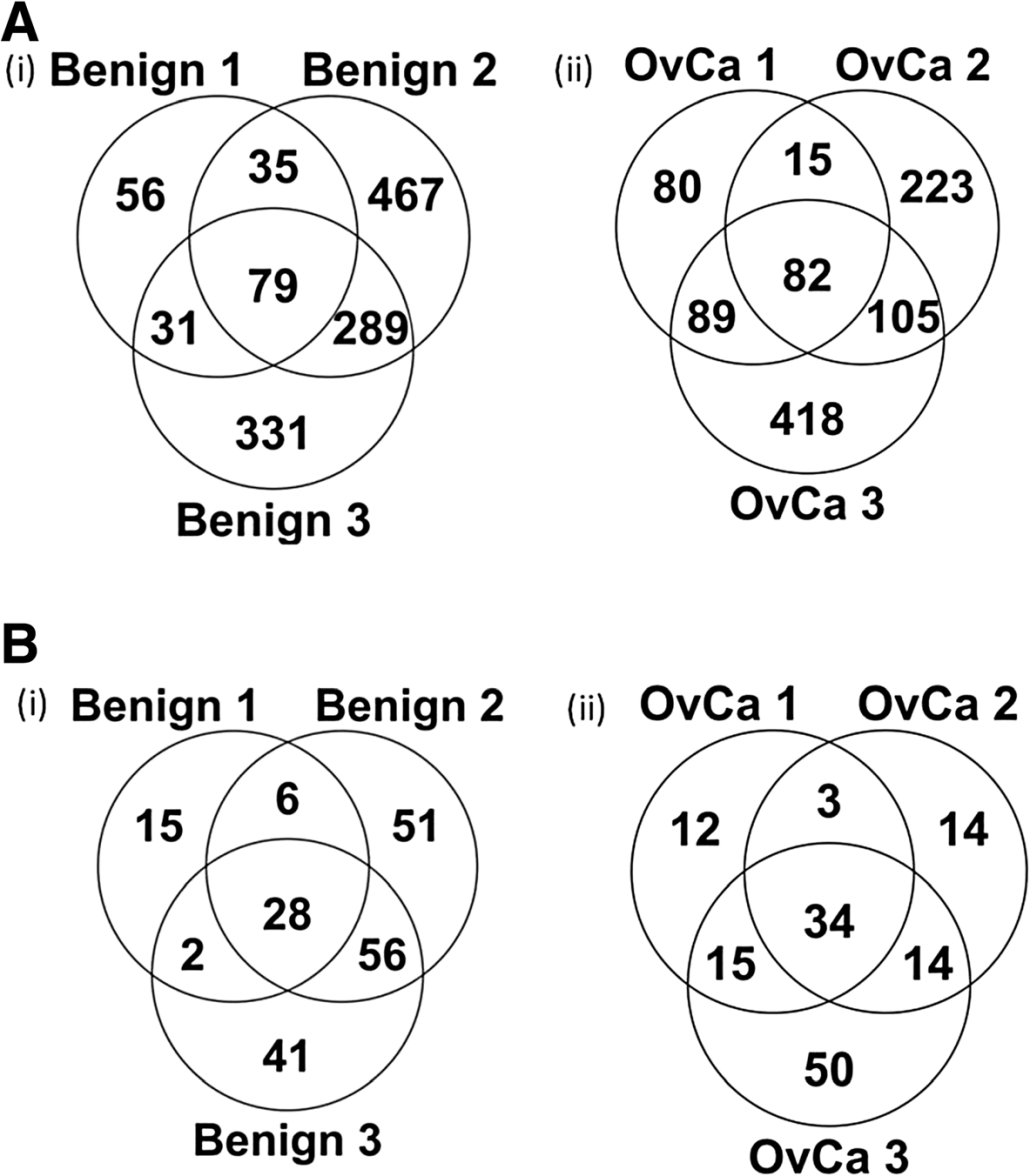
**Biological repeatability at the peptide (A) and protein (B) level across the 3 benign and 3 ovarian cancer (OvCa) ascites samples.** Overlap of unique peptides **(A)** and corresponding parent proteins **(B)** identified amongst (i) the three benign control ascites samples and (ii) the three OvCa ascites samples. These samples represent biological replicates (not technical replicates). Each sample was analyzed in singleton.

Figure 3 (A) and (B) shows the overlap of identified peptides and proteins, respectively, within the group of 3 OvCa samples and the group of 3 benign samples. About 37-39% of peptides and 50-58% of proteins were found in at least 2 samples. These data are to be expected if we consider the biological heterogeneity of these samples (they all came from different individuals).

### Overlap with previously-delineated OvCa ascites proteome

Kuk et al. previously mined the proteome of OvCa ascites, identifying 445 proteins by combining mass spectrometry data from multiple size exclusion and fractionation protocols [18]. Of the 142 parent proteins we found in OvCa ascites (including the 23 proteins common to the control cohort) (Figure 2), 73 were also found by Kuk et al. This significant overlap (51%) corroborates our original hypothesis that many endogenous peptides are derived from proteins already present in the mid- to high-molecular-weight region of ascites (>10 kDa). Given that our peptides were endogenous degradation products, while Kuk et al.’s peptides resulted from trypsin digestion of large proteins, we expected to see little overlap at the peptide level. Indeed, only 24 of 4585 total peptides identified were common to both studies. This lends support to our experimental protocol’s ability to identify novel, endogenous peptides.

### Overlap with existing OvCa serum proteome studies

Fredolini et al. compared the low-molecular-weight serum proteome of 20 early-stage epithelial ovarian cancer patients to the corresponding proteome of 20 patients with benign gynecological conditions [2]. From our OvCa ascites samples, we identified 12 of the 59 serum proteins deemed overexpressed in OvCa by Fredolini et al. Since serum is an entirely different biological fluid, this relatively low rate of overlap (20.3%) is unsurprising. Another study, by Lopez et al., examined the serum proteome of 110 healthy individuals and 430 OvCa patients (stages I-IV) [10]. The authors identified 160 proteins overexpressed in OvCa patients compared to healthy controls. Our study identified 13 of these proteins, which represents an overlap of 8.1%. Two of these common proteins, transthyretin and apolipoprotein A1, are components of the five marker, FDA-approved OVA1 panel for discriminating cancerous from non-cancerous ovarian masses [12].

### Overlap with existing urine peptidomes

Smith et al. (manuscript submitted) delineated the peptidome of 6 late-stage OvCa and 6 healthy urine samples (each processed in triplicate), identifying 3646 peptides from 514 proteins in the 6 ovarian cancer samples. We identified 64 of these proteins in our 3 ovarian cancer ascites samples, as well as 29 common peptides. The very low overlap at the peptide level (less than 1%) may reflect the fluid-specificity of both proteins and proteases. Smith et al. may have found entirely different peptides in urine either because different proteases act in the renal system and in urine, or because different peptides make their way into the urine as compared to ascites. Either way, this preliminary finding may hint at differences in how peptides are processed by the circulatory and renal systems at different stages and in different fluids. Moreover, peptides are considered unique by Scaffold if they differ by as little as one amino acid, and this stringency could also limit the number of unique peptides we report as being common to both peptidomes. The 29 common peptides derive from 10 proteins, the majority of which are high-abundance proteins.

Siwy et al. compiled a human urinary peptide database from healthy individuals [19]. Of the 114 proteins they identified, we found 35. At the peptide level, we only found 12 common peptides. This result may further corroborate the fluid-specificity of proteins and proteases.

A six-way comparison of identified proteins in the current study and in each of the studies cited above is provided in Additional file 4.

### Ovarian cancer-specific candidates

We filtered our list of identified proteins to select only those proteins for which we identified peptides exclusively (or almost exclusively) in the three ovarian cancer samples (Table 1). Common high-abundance serum proteins were excluded, with the exception of haptoglobin, whose differing proteolytic cleavage pattern between cancerous and benign samples merits further study (as outlined below). We arrived at a final list of 12 proteins we deem most worthy of further verification and validation, separated into three categories: those previously studied as ovarian cancer biomarkers, those previously studied in ovarian cancer (including in gene expression studies) but not as biomarkers, and those never before studied in ovarian cancer.

**Table 1:** List of top protein/peptide candidates identified in ovarian cancer ascites fluid

**Parent protein**	**# of peptides (OvCa)**	**# of peptides (Benign)**
Retinol-binding protein 4	1	0
Serum paraoxonase/arylesterase 1	14	0
Talin-1	2	0
Isoform 1 of ficolin-3	1	0
Vitronectin	31	7
Apolipoprotein F precursor	12	1
Isoform 1 of Vitamin D-Binding protein	21	0
Histidine-rich glycoprotein	16	0
Transketolase (highly similar cDNA)	4	0
Mannosidase	2	0
EGF containing fibulin-like extracellular matrix protein 1	3	0
Haptoglobin	47	0

Number of total unique peptides identified (for each candidate parent protein) in the three ovarian cancer ascites samples (OvCa) and the three benign ascites samples. Thresholds for high-confidence peptide identifications were set to ensure that false discovery rate (FDR) at the peptide level was close to 1%.

#### Candidates previously studied as ovarian cancer biomarkers

Out of our top 12 candidates, five proteins have been previously examined as ovarian cancer biomarkers. Below, we briefly describe each of these five proteins and their association with OvCa. We identified endogenous peptide fragments of *Retinol-binding protein 4,* which has been previously studied in ovarian cancer (using proteomic 2-D electrophoresis), and was suggested to have potential as a diagnostic or prognostic marker at the protein level [20]. Interestingly, the cited study found decreased, not increased, levels of the protein in the sera of epithelial ovarian cancer patients.

Similarly, a previous study found decreased activity of *Serum paraoxonase and arylesterase* (another one of our peptide candidates) in the sera of ovarian cancer patients [21]. Several polymorphisms of the corresponding gene, PON1, have been associated with increased risk of ovarian cancer [22,23].

Our third candidate was *Vitamin D-Binding Protein.* A recent study found that serum levels of this protein in ovarian cancer did not differ significantly from levels in healthy controls or individuals with benign conditions [24]. We confirmed this upon preliminary verification using an ELISA and found that serum levels of Vitamin D-binding protein showed little ability to discriminate patients with ovarian cancer from healthy controls (data not shown).

Our candidate list includes peptides derived from an isoform of *ficolin-3*. Serum ficolin-3 was previously found to be overexpressed in the sera of ovarian cancer patients [25].

Finally, the potential utility of *histidine-rich glycoprotein* to discriminate ovarian cancer has been previously reported [26]. A two-marker panel of this protein along with corticosteroid-binding globulin (not found in our study) showed comparable performance to CA-125 in differentiating early stage ovarian cancer from normal controls [26].

Importantly, none of the above mentioned five proteins have been examined at the peptide level in ovarian cancer. The endogenous peptides corresponding to each protein may well have higher diagnostic sensitivity or specificity than those reported thus far. Future studies along these lines are warranted.

#### Candidates previously studied in ovarian cancer

Four of our top candidates have been previously examined with respect to OvCa but not as biomarkers. For example, *vitronectin* is an important factor in the progression of ovarian cancer, and has been previously assayed in ascites fluid [27]. More importantly, cleavage of vitronectin and fibronectin into small fragments (mediated by MMP-2) is a key initiator of metastasis in ovarian cancer [28]. The generation of these fragments during disease progression lends support to our finding of vitronectin-derived peptides in ovarian cancer samples but not in the benign controls. Endogenous peptide levels of vitronectin in biological fluids requires further investigation.

*Talin-1* has been previously implicated in serous ovarian carcinoma, particularly in its development and progression of metastasis [29]. A recent study found that a specific microRNA, miR-9, which directly targets talin-1, acts as a tumor suppressor in ovarian cancer [29].

*EGF containing fibulin-like extracellular matrix protein 1* was identified as overexpressed in chemoresistant OvCa tissue using quantitative proteomics [30].

Finally, *transketolase* is involved in the pentose phosphate shunt and aerobic glycolysis, the primary source of energy for most fully transformed carcinomas [31]. Expression of Transketolase-like protein 1 (TKTL1), a similar protein to transketolase, was elevated in serous papillary ovarian adenocarcinomas [31]. The study found that expression of TKT1 was also shown to predict poor prognosis.

None of the above mentioned four candidates have been investigated as possible biomarkers in ovarian cancer, at either the protein or peptide levels.

#### Candidates not previously studied in ovarian cancer

*Alpha-mannosidase* has been previously identified in the peritoneal fluid of patients with gynecologic cancers and pelvic inflammatory disease, but has never been studied specifically in ovarian cancer [32]. Lastly, there is no existing literature on *Apolipoprotein F* in ovarian cancer.

#### Haptoglobin: revisiting a high-abundance protein at the peptide level

**Figure 4:**
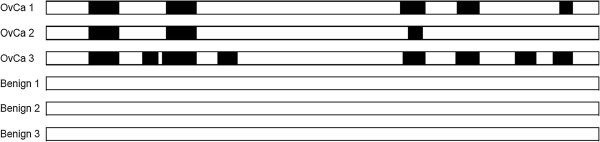
**Peptides identified from haptoglobin.** A schematic representation of the haptoglobin protein. The highlighted areas reveal the peptides identified per sample. No peptides were identified in the benign samples.

*Haptoglobin* is a high-abundance serum protein, and has been extensively studied for use as an OvCa marker [33,34], but has never before been studied at the peptide level. In this study, peptides originating from haptoglobin were found exclusively in the three ovarian cancer samples. We identified 47 such peptides in total. Figure 4 displays a schematic representation of identified peptides from the haptoglobin protein in the three OvCa samples, shaded to highlight identified peptide sequences along the length of the entire protein. We noticed that there were five conserved sequences common to at least two of the OvCa samples. Peptides sharing this common “protein core” may have diagnostic utility and merit further investigation. No peptides from haptoglobin were identified in the three benign samples.

#### Summary

Most of the 12 candidates discussed above have either been studied as OvCa biomarkers at the protein level or are implicated in OvCa pathogenesis. Studying some candidates via selected reaction monitoring quantitative mass spectrometry at the peptide level may reveal novel OvCa biomarkers. Assays for these peptides in various biological fluids are currently under development.

## Conclusion

In this study, we employed a new experimental workflow to elucidate the peptidome of ovarian cancer ascites fluid. Our strategy identified over 2000 endogenous peptides and is, to the best of our knowledge, the first peptidomic study of ovarian cancer ascites. Comprising 777 unique peptides found exclusively in OvCa ascites, our work is the first step towards identifying novel OvCa-specificpeptides. Indeed, each of our 12 promising candidates requires further verification and validation using quantitative selected reaction monitoring assays on a larger OvCa cohort. The fragmentation pattern of haptoglobin in OvCa and its corresponding degradome also merit further investigation.

## Methods

### Sample collection and preparation

Ascites fluid was obtained from 3 late-stage serous epithelial ovarian cancer patients and 3 patients with cirrhosis of the liver, after written informed consent and institutional ethics board approval. REB approval is from University Health Network Research Ethics Board. For each patient, approximately 1 L of ascites fluid was collected via paracentesis. The samples were aliquoted into 50 mL tubes, centrifuged, and the supernatant was isolated and frozen in −80°C immediately (approximately 3 hours from the time of sample collection to freezing the supernatant). Samples were stored at −80°C until further analysis.

Before sample preparation, ascites samples were thawed and vortexed to re-suspend any precipitate. All specimens (cases and controls) were processed in parallel, using the same lot of reagents and columns, to minimize bias in sample preparation. The samples were centrifuged at 17 000 g for 10 minutes, then supernatants were diluted in phosphate buffered saline (Multicell PBS 1×) to bring the total protein concentration down to 1 mg/mL for each sample (this equated to approximately 20 μL of starting volume for each biological replicate). A final concentration of 2 mM of dithiothreitol from Sigma-Aldrich was added and the samples were incubated at room temperature before a final concentration of 4 mM iodoacetamide was added. Fifteen millilitres of the ascites fluid was then concentrated using a Vivaspin 20 mL ultrafiltration spin column with a 10 kDa cutoff membrane (Sartorius Stedim Biotech), according to the manufacturer’s instructions (columns were pre-equilibrated with water before use).

The flow-through obtained after concentration was acidified by drop-wise addition of formic acid to pH 3. The samples were then passed through a hydrophilic-lipophilic-balanced reversed-phase cartridge (Oasis HLB). The cartridge (1 cc (30 mg); Waters cat# WAT094225) was pre-equilibrated with 1 mL 90% acetonitrile (ACN), 0.1% formic acid. The cartridge was then washed with 5 mL of Buffer A (5% ACN, 0.1% formic acid), before application of the acidified sample. Following the loading of sample, the cartridge was washed again with 5 mL of Buffer A. Peptides were eluted with the addition of 0.5 ml of 60% ACN, 0.1% formic acid. The sample was reduced to a volume of approximately 200 μL via lyophilization.

### Strong cation exchange chromatography

The sample was diluted in mobile phase A (0.26 M formic acid in 5% ACN) to bring the starting volume up to 1 mL prior to injection into a PolySULFOETHYL A column with a 200-Å pore size and diameter of 5 μm (The Nest Group, Inc.) containing a hydrophilic, anionic polymer (poly-2-sulfethyl aspartamide). A 1 hour separation was performed on an HPLC system (Agilent 1100) using a mobile phase B containing 0.26 M formic acid in 5% ACN and 1 M ammonium formate. The eluate was monitored at a wavelength of 280 nm. Seven fractions per sample were collected at a flow rate of 200 μL/min.

### Mass spectrometry

The resulting fractions were desalted on an Omix C18 pipette tip (Varian) and eluted in 5 μL of buffer B (70% acetonitrile, 0.1% formic acid). After elution, we added 80 μL of buffer A (0.1% formic acid) to each sample before loading 40 μL of each onto a 2-cm C18 trap column, packed with Varian Pursuit (5 μm C18), using the EASY-nLC system (Proxeon Biosystems). Peptides were eluted from the trap column onto a resolving 5-cm analytical C18 column packed with Varian Pursuit (3 μm C18) with an 8 μm tip (New Objective). This LC setup was coupled online to an LTQ-Orbitrap XL (Thermo Fisher Scientific) mass spectrometer using a nanoelectrospray ionization source (Proxeon Biosystems). Each fraction underwent a 54-min gradient, and eluted peptides were subjected to 1 full scan (350–2000 m/z) in the Orbitrap at 60 000 resolution, followed by top 6 data-dependent MS/MS scans in the linear ion trap. With the use of charge-state screening and preview mode, unassigned charge states were rejected.

### Data analysis

Raw files were used to generate Mascot Generic Files (MGF) through extract_msn on Mascot Daemon (version 2.2.2). Once generated, MGFs were searched with X!Tandem (Global Proteome Machine Manager; version 2006.06.01) to confer peptide identifications. Searches were conducted against the non-redundant Human IPI database (v.3.71) which contains a total of 173,490 forward and randomized protein sequences and using the following parameters for GPM: no enzyme ([X]|[X]) cleavages, 50 missed cleavage sites allowed, 7 ppm precursor ion mass tolerance, 0.4 Da fragment ion mass tolerance, fixed modifications of carbamidomethylation of cysteines, and variable modification of oxidation of methionines (oxM). The X!Tandem (XML files) were then integrated through the Scaffold 2 software (version 2.06; Proteome Software Inc., Portland, Oregon). False-discovery rates were calculated as the number of peptides identified by the randomized reverse database divided by the total number of identified peptides.

## Abbreviations

OvCa: Ovarian cancer; CA125: Carbohydrate antigen 125; GPM: Global proteome machine; LMW: Low-molecular-weight; TKTL1: Transketolase-like protein 1.

## Competing interests

The authors have declared no conflict of interests.

## Authors' contributions

AB, FL and CRS carried out the experimental protocol and data analysis. EPD and VK participated in the design of the study and aided in data analysis. All authors participated in the writing of the manuscript and have read/approved the final draft.

## Supplementary Material

Additional file 1 Detailed information on peptides (A) and proteins (B) identified in all 6 studied samples. Click here for file

Additional file 2 Detailed information on the 777 peptides (A) and 59 protiens (B) identified only in the three OvCa samples. Click here for file

Additional file 3 A detailed spectrum report of all identified peptides (including data about peptide modifications). Click here for file

Additional file 4 List of overlapping proteins with other publications. Click here for file
